# Exploring distinct default mode and semantic networks using a systematic ICA approach

**DOI:** 10.1016/j.cortex.2018.12.019

**Published:** 2019-04

**Authors:** Rebecca L. Jackson, Lauren L. Cloutman, Matthew A. Lambon Ralph

**Affiliations:** a MRC Cognition & Brain Sciences Unit, University of Cambridge, Cambridge, UK; b Neuroscience and Aphasia Research Unit (NARU), Division of Neuroscience & Experimental Psychology (Zochonis Building), University of Manchester, Manchester, UK

**Keywords:** Connectivity, Default mode network, Independent component analysis, Resting-state networks, Semantic cognition, DMN, default mode network, FC, functional connectivity, ICA, independent component analysis, IFG, inferior frontal gyrus, IPL, inferior parietal lobe, mPFC, medial prefrontal cortex, pMTG, posterior middle temporal gyrus, RS, resting-state, RSN, resting-state network, vATL, ventral anterior temporal lobe

## Abstract

Resting-state networks (RSNs; groups of regions consistently co-activated without an explicit task) are hugely influential in modern brain research. Despite this popularity, the link between specific RSNs and their functions remains elusive, limiting the impact on cognitive neuroscience (where the goal is to link cognition to neural systems). Here we present a series of logical steps to formally test the relationship between a coherent RSN with a cognitive domain. This approach is applied to a challenging and significant test-case; extracting a recently-proposed semantic RSN, determining its relation with a well-known RSN, the default mode network (DMN), and assessing their roles in semantic cognition. Results showed the DMN and semantic network are two distinct coherent RSNs. Assessing the cognitive signature of these spatiotemporally coherent networks directly (and therefore accounting for overlapping networks) showed involvement of the proposed semantic network, but not the DMN, in task-based semantic cognition. Following the steps presented here, researchers could formally test specific hypotheses regarding the function of RSNs, including other possible functions of the DMN.

## 1 Introduction

It is increasingly recognised that higher cognitive functions are not localised to a single cortical region but rather reflect coordinated interactions across a distributed brain network. As such, assessments of functional connectivity (FC) are integral to understanding how regions work together to support complex cognition. Significant interest surrounds resting-state networks (RSNs); groups of regions shown to co-activate without performance of an explicit task, thought to reflect the intrinsic connectivity associated with different cognitive domains (Frackowiak and Markram, 2015, Van Essen et al., 2013). To use RSNs to increase understanding of a cognitive domain, researchers must identify the coherent network involved in a specific function of interest. However, often the methods used are inadequate to achieve this aim, failing to ensure that the sets of identified regions constitute coherent networks and providing no formal demonstration of the link between a RSN and its associated cognitive function.

**Fig. 1:**
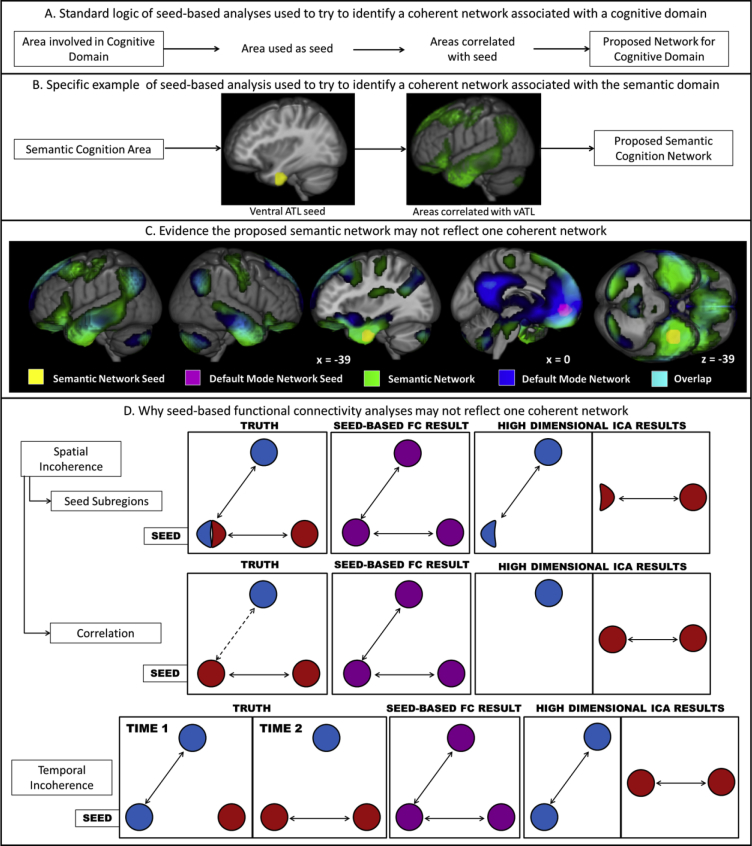
Identifying domain-related RS connectivity with seed-based FC analyses. A. One process used to identify connectivity associated with a domain of interest; a key area responsible for that process is used as a seed and all voxels that have a time series correlated with it are identified. This set of regions is assumed to form a network responsible for the domain of interest. B. An example of this logic applied to the semantic domain. The ventral anterior temporal lobe (vATL) plays a key role in semantic cognition. The areas connected to the vATL (shown in yellow) during rest were identified using a seed-based FC analysis in Jackson, et al. (Jackson, Hoffman, Pobric, & Lambon Ralph, 2016). These are shown in green and have a voxel-level significance threshold of .001 and an FWE-corrected critical cluster level of .05. These regions were proposed to form a network for semantic cognition. C. Overlap between the proposed semantic network and the DMN. The DMN (shown in blue) was determined as the functional connectivity of a mPFC seed (shown in violet; MNI coordinates: −1 47 -4; Fernandez-Espejo et al., 2010, Fox et al., 2005, Mennes et al., 2010, Takeuchi et al., 2013, Viviani et al., 2011, Whitfield-Gabrieli et al., 2009) and is overlaid on the proposed semantic network (green). The vATL seed used to identify the semantic network is shown in yellow. The peak areas are provided in Table 1. Overlap (cyan) may be seen in lateral and ventral ATL, ventral and dorsal mPFC, angular gyrus and a small region of the precuneus. Both networks have a voxel-level significance threshold of .001 and an FWE-corrected critical cluster level of .05. The high level of overlap between the two networks suggests that their relationship should be assessed and the proposed semantic network may not be one coherent network. D. Cartoons of possible scenarios in which a seed-based FC result may not reflect one coherent network yet a high-dimensional ICA result would. In all cases the underlying truth is displayed on the left followed by the seed-based FC result and the ICA on the right. The scenarios include spatially incoherent results, either due to a seed region including multiple functional subregions or due to the region being correlated with a distinct network, and temporally incoherent results due to the seed region being involved in multiple networks over time.

One common method to identify a RSN that may relate to a domain of interest is seed-based FC analysis which identifies all regions that have a time-series correlated with that of a seed region-of-interest (Biswal, Yetkin, Haughton, & Hyde, 1995). By using a seed region with a known function, researchers seek to identify a network associated with that function (Fig. 1A&B). However, there are at least three alternate situations whereby regions identified as connected to the seed may not form a coherent network either over time or space (see Fig. 1D). Firstly, the result will not be temporally coherent if the same area is engaged in multiple networks at different times, reflecting distinct cognitive processes (Geranmayeh et al., 2012, Geranmayeh et al., 2014, Leech et al., 2011). Secondly, discrete networks may involve different parts of the chosen seed region. The third possibility is that networks may be entirely spatially separable and functionally distinct but exhibit RS correlation because much of their time-course is similar. Positive and negative relationships between distinct RSNs are well documented (Fox et al., 2005, Humphreys et al., 2015). The latter two possibilities would cause a failure to identify a spatially coherent network, whereas the first represents an inability to define a temporally coherent network. An additional issue with seed-based FC analyses is that the artificial inflation of relationships between nearby regions, caused by pervasive effects of motion, results in a loss of spatial specificity (Power et al., 2014, Power et al., 2015, Van Dijk et al., 2012).

Unlike seed-based FC analyses, model-free approaches can delineate distinct, coherent RSNs. Independent component analysis (ICA) is a data-driven multivariate technique whereby variance in the BOLD signal over space and time is separated into independent ‘components’, each consisting of a spatial map and time-course of activity (Calhoun et al., 2001, Geranmayeh et al., 2014, McKeown and Sejnowski, 1998). Any ICA utilising a relatively large number of components may be referred to as a ‘high-dimensional ICA’. High-dimensional ICA has been successful in separating spatially overlapping networks within task and RS data, resulting in spatially and temporally coherent networks (see Fig. 1D; Beckmann et al., 2005, Calhoun et al., 2008, Geranmayeh et al., 2012, Geranmayeh et al., 2014, Smith et al., 2009). Additionally, the identification of artefact-related components, allows the effect of motion to be reduced in ICA compared to seed-based FC analyses (Griffanti et al., 2014, Thomas et al., 2002).

Despite the advantages of ICA over seed-based analyses it remains underemployed, particularly when investigators wish to identify FC related to a specific domain of interest. This is because ICAs identify many components (typically 20–100 components) making interpretation complex (de la Iglesia-Vaya, Molina-Mateo, Escarti-Fabra, Kanaan, & Marti-Bonmati, 2013). By definition, all RS analyses suffer from an inherent detachment from cognition and thus RS connectivity alone provides no information on network function. Despite this lack of functional information, however, researchers are often willing to interpret a single seed-based result as related to their domain of interest, yet unable to provide interpretations of multiple networks from ICA. Thus, whilst ICA allows coherent RSNs to be identified, it may exacerbate the problem of functional interpretation through an overwhelming number of results.

RS ICA does not provide any formal link to the function of a network. However, a great deal of research using diverse methodologies has demonstrated the similarity between networks present in resting and task states (e.g., Bertolero et al., 2015, Bzdok et al., 2016, Cole et al., 2014, Crossley et al., 2013, Krienen et al., 2014, Laird et al., 2011, Smith et al., 2009, Yeo et al., 2015), albeit in the context of secondary state-dependent connectivity changes (Ganger et al., 2015, Krienen et al., 2014). This research suggests that RSNs perform similar functions in rest and task states. Therefore, identification of a RSN in task data would allow investigation of its functional characteristics. This is rarely done in order to determine the function of a specific RSN, although some recent novel methodologies have adopted this approach (e.g., Lorenz et al., 2018). In standard RS ICA investigations, components are often labelled by eye based on functional interpretation of the constituent regions (Beckmann et al., 2005, Jafri et al., 2008). Attempts to determine the function of a specific RSN have usually focussed on visual comparison between the RSN and the areas identified in univariate analyses or meta-analyses of task data (Binder et al., 2009, Buckner et al., 2008, Mars et al., 2012, Murphy et al., 2018). Comparing the network extent to results of large meta-analyses, including online meta-analytic techniques such as the BrainMap database (Laird et al., 2011, Margulies et al., 2016, Smith et al., 2009) has benefits in terms of power and specificity in contrast to a single study. However, task results are baseline dependent and difficulty differences are known to consistently identify the same areas as known RSNs (Gilbert et al., 2012, Vincent et al., 2008). Therefore, univariate analyses including meta-analyses could reflect RSNs without those networks being responsible for the task. Additionally, these analyses identify areas and not coherent networks. This means it is not clear how to interpret differences in extent or missing areas between the RSN and task results, particularly as many areas are involved in multiple networks. Meta-analytic approaches do not solve the inherent problem that the total difference in activation between two conditions is inadequate to demonstrate the functional involvement of a specific network. Therefore, comparison to task activation may be best seen as generating hypotheses as to the function of a RSN which could be tested by undertaking formal analyses of the role of the coherent network in task data.

One simple way to formally test the function of a RSN would be to take its spatial extent as a volume-of-interest and assess its activity in different task conditions. This approach is very precise about the areas involved and can use the spatially coherent networks identified in the RS ICA, however, the temporal information is lost. Thus, if an area is involved in multiple networks, under this temporally-insensitive VOI approach, they will not be correctly separated. In order to determine the function of the network more accurately, it is necessary to assess the cognitive signature of the spatially and temporally coherent network itself and not merely the activity of the constituent regions. Thus, we demonstrate a series of logical steps designed to avoid these issues. Coherent RSNs of interest may be identified using an ICA and template matching. Independent task data may be used to identify the same coherent networks using a separate ICA and quantification of the overlap between these networks. The proposed function of this network of interest can then be formally assessed within the task data. Unlike the standard logic of a task ICA, the aim here is to identify the coherent RSN of interest to determine its function, not merely to identify all networks associated with a task.

As a pertinent test-case to demonstrate this widely applicable process, we investigated the ability to extract a recently proposed semantic RSN and assess its relation with the default mode network (DMN). As reviewed briefly below, this is a suitably demanding test-case for the following reasons: (i) semantic processing is supported by a distributed network; (ii) these regions have been shown to be functionally connected in RS data with seed-based FC analyses (Jackson et al., 2016, Pascual et al., 2013) yet this network has not been formally linked to semantics; (iii) there is a high level of overlap between this proposed RSN and the DMN (Buckner et al., 2008, Greicius et al., 2003); (iv) multiple theories of DMN function have been posited including an inherent relationship with semantic cognition (e.g., Binder et al., 1999, Binder et al., 2009), yet these theories remain to be formally tested.

Representation of multimodal conceptual knowledge, relies upon a multimodal hub within the anterior temporal lobe (ATL), interacting with modality-specific regions (Lambon Ralph, 2014, Lambon Ralph et al., 2017, Patterson et al., 2007). Inferior frontal gyrus (IFG), medial prefrontal cortex (mPFC), posterior middle temporal gyrus (pMTG) and inferior parietal lobe (IPL) are critical for the controlled retrieval and manipulation of semantic knowledge, or “semantic control” for short (Jefferies, 2013, Lambon Ralph, 2014, Noonan et al., 2013, Patterson et al., 2007). Signal loss and distortion within vital inferior temporal and frontal regions has impaired our ability to assess semantic-related FC during tasks and RS data (Embleton et al., 2010, Visser et al., 2010a, Wig et al., 2014, Zuo et al., 2012). Newly-developed techniques (distortion-corrected spin echo and dual echo fMRI) have reduced these artefacts and improved extraction of ATL signal (Embleton et al., 2010, Halai et al., 2014, Jackson et al., 2015, Visser et al., 2010a), and have recently been used to collect RS data (Jackson et al., 2016). From these active and RS fMRI data, connectivity has been demonstrated between the ATL and other regions responsible for multimodal semantic processing, including the IFG, mPFC, pMTG and IPL (Jackson et al., 2016). Many of these regions are also identified in the DMN which includes mPFC, posterior cingulate cortex and precuneus (Buckner et al., 2008, Greicius et al., 2003). Additional involvement of medial temporal lobe, angular gyrus and lateral ATL is sometimes identified (Andrews-Hanna et al., 2010, Buckner et al., 2008). These regions have been shown to deactivate during some but, importantly, not all tasks (Buckner et al., 2008, Fox et al., 2005, Spreng, 2012). To formally demonstrate the overlap with the semantic network, a seed-based DMN was identified within the same data using a standard mPFC seed (displayed in Fig. 1C.; Fernandez-Espejo et al., 2010, Fox et al., 2005, Mennes et al., 2010, Takeuchi et al., 2013, Viviani et al., 2011, Whitfield-Gabrieli et al., 2009; for details see Methods). The overlap between the two seed-based networks is displayed in Fig. 1C.

Although thousands of papers discuss semantics or the DMN, very few address the potential relationship between these networks (for reviews see Binder and Desai, 2011, Buckner et al., 2008, Lambon Ralph, 2014, Lambon Ralph et al., 2017). There may be two coherent and distinct networks that are either (i) spatially overlapping yet discriminable over time or (ii) spatially distinct but inadequately separated by the seed-based FC analysis. Alternatively, there may only be one coherent RSN. The function of the networks must also be considered and the proposed relation of the ‘semantic network’ to semantic cognition must be verified. The DMN has also been argued to relate to semantics as sufficiently difficult tasks involving semantic memory may not show the expected deactivation in DMN regions (Binder et al., 1999, Binder et al., 2009, Shapira-Lichter et al., 2013, Wirth et al., 2011). This has led to the suggestion that a process occurs frequently during ‘rest’ which relies, at least in part, upon semantic processing (Binder et al., 2009, Humphreys et al., 2015). However, this result has not been widely observed and might instead relate to differences in task/item difficulty (Binder et al., 1999, Shapira-Lichter et al., 2013, Wirth et al., 2011). Visual comparison of the DMN to the results of meta-analyses have been argued to show that it is responsible for semantics (Binder et al., 2009), episodic memory (Buckner et al., 2008) and social cognition (Mars et al., 2012). However, these hypotheses require formal testing at the level of the coherent DMN. In order to interpret the existing results on the semantic network and the DMN, therefore, we identified the relevant coherent networks and then assessed their cognitive signature, in particular their relation to semantic cognition. We assess this relation using a standard verbal semantic judgement task. Although it may be hypothesised that the DMN performs a specific form of semantics (e.g., ‘complex’ or ‘social’ semantics), we limit our assessment and interpretation to general semantics for the present test case as this has previously been hypothesised to be the function of both the proposed semantic network and the DMN.

## 2 Materials & methods

### 2.1 Participants

Resting-state scans were collected for 78 participants (57 female, age range 18–42, average age 24.71 years, standard deviation 5.49 years; analyses using this dataset were previously reported in Jackson et al., 2018, Jackson et al., 2016, Jung et al., 2016), 24 of whom also completed the semantic judgement task reported previously (Jackson et al., 2015; 15 female, age range 20–42, average age 25.48 years, standard deviation 6.49 years). All had normal or corrected-to-normal vision and were strongly right handed [laterality quotient on the Edinburgh Handedness Inventory (Oldfield, 1971); minimum 50, average 85.85, standard deviation 14.91]. All participants gave written informed consent and the study was approved by the local ethics board.

### 2.2 Imaging protocol

The imaging parameters used to acquire the RS and task data were identical. Scanning was conducted using a Phillips Achieve 3.0T system with a 32 channel SENSE coil with a sense factor of 2.5. A structural reference was obtained with an in-plane resolution of .938 and slice thickness of 1.173. Whole brain coverage was obtained with a field of view of 240 × 240 mm, which was tilted up to 45° off the AC-PC line to reduce the effect of ghosting on the temporal pole. The resolution matrix was 80 × 80 with a reconstructed voxel size of 3 mm and slice thickness of 4 mm. The flip angle was 85°. This resulted in a TR of 2.8. Noise cancelling Mk II + headphones were worn inside the scanner (MR Confon, Magdeburg, Germany).

In order to reduce signal dropout in inferior temporal and frontal cortex, including ATL regions critical for semantic cognition, a dual gradient echo EPI technique was employed. Data were acquired in parallel for a short (12 ms) and a long (35 ms) echo time and summed linearly in order to preserve signal in problematic regions whilst maintaining contrast sensitivity throughout the brain (Halai et al., 2014, Poser and Norris, 2007, Poser and Norris, 2009, Poser et al., 2006). For further details and TSNR maps see Jackson et al. (2016).

### 2.3 Overlap measure

The proposed semantic network identified based on seed-based resting-state analyses in Jackson et al. (2016) was used as a template to identify a coherent semantic network in the ICA results. Although this should not be seen as a ‘gold standard’ of the network due to the problems inherent in seed-based network analyses, it is the current best estimate of the proposed network's spatial configuration. This is considered sufficient as a means to identify the network, yet does not presuppose that no differences will be identified. In order to demonstrate the overlap between the identified semantic network and the DMN and to have a comparable result to use as a DMN template, the DMN was identified using a seed-based analysis in the same resting-state data. The methodology was identical to Jackson et al. (2016) except a seed region within the mPFC (MNI coordinates: −1 47 -4) was used, as in a number of prior studies attempting to identify a DMN (Fernandez-Espejo et al., 2010, Fox et al., 2005, Mennes et al., 2010, Takeuchi et al., 2013, Viviani et al., 2011, Whitfield-Gabrieli et al., 2009). A spherical 10 mm ROI was constructed around this location. The ROI location and the results of this analysis are shown in Fig. 1C and Table 1.

**Table 1:** Significant clusters of the default mode network during the resting-state, determined by functional connectivity to an *a priori* medial prefrontal cortex seed.

Cluster Region	Cluster extent (voxels)	Max z value	P value (FWE corrected)	Peak MNI Coordinate		
				X	Y	Z
mPFC, precuneus, MCC, PCC, MTL, lateral & ventral ATL	14825	>8	>.001	0	48	−6
				6	45	0
				−6	−57	21
L AG	646	>8	>.001	−48	−69	36
R AG	453	>8	>.001	51	−63	33
Cerebellum	414	>8	>.001	48	−66	−42
				27	−81	−33
				21	−90	−39
Cerebellum	311	7.61	>.001	6	−57	−45
				−6	−57	−42
Cerebellum	235	6.92	>.001	−30	−81	−36
				−45	−75	−42
				−18	−90	−39

Clusters significant at .001 after FWE correction. Largest 3 peaks listed per cluster. L = left. R = right. MTL = medial temporal lobe, PCC = posterior cingulate cortex, MCC = mid cingulate cortex, CG = central gyrus.

The RS ICA group-level components were compared to the ATL-seeded semantic network and the mPFC-seeded DMN network as well as a publically available DMN template (Shirer, Ryali, Rykhlevskaia, Menon, & Greicius, 2012) to ensure that the DMN identified reflected the consensus in the literature. The task components of interest were identified using the RS components of interest as templates. Seed-based networks and group-level ICA components were thresholded at a voxel-level significance of .001 and an FWE-corrected critical cluster level of .05 and then binarised. As the spatial map for each ICA component is constructed by assessing whether each voxel is significantly active in the individual participant's component results, it is a standard statistical map and can therefore be subjected to thresholding and binarisation. The degree of overlap of each component was then quantified using the Jaccard similarity coefficient between the two images (Jaccard, 1912). Thus, the number of voxels active in both the template and component images was divided by the total number of voxels active in either the template or the component image or both. The code used to obtain the Jaccard similarity coefficients per component is provided in the Supplementary Materials.

The Jaccard similarity coefficient (J) is a measure of overlap ranging from 0 (no overlap of active voxels) to 1 (full overlap of both active and inactive voxels). This means the components are assessed in terms of how well they match the extent of the template and have a lower overlap score if they miss areas in the template or if they have areas not included in the template. Thus, the Jaccard coefficient provides a single measure of the fit between the component and the template. The Jaccard similarity coefficient has been used previously to assess similarity across brain activation maps (James et al., 2016, Karahanoğlu and Van De Ville, 2015, Maitra, 2010, Ryyppö et al., 2018). The overlap assessment was a two-step procedure. In the first step all components that reached a threshold of J = .15 were identified. This is a relatively low threshold in order to allow for the identification of all relevant components in situations where the overlap with each may be low, for instance, if the network has split in to multiple components or if the template is not very accurate. This may be the case in the current situation as the seed-based templates may be inaccurate and the task context may cause the networks to split in to multiple components. If multiple components are identified in this first step, a second step was performed where the combination of these components are assessed. In this case the first component (the best fit) can be combined with the next best fit and the overlap between this combined image and the template calculated. If this gives a numerical improvement, both the components can be considered to relate to the template network. This process can be repeated for any number of components that each reached the similarity threshold. If adding these components together leads to a numerical improvement, then all are identified as part of the network.

This two-step process identifies all components that may be relevant and then assesses whether it is best to think of multiple components as related to the template network or not. If, for instance, a network has split in two and both parts are somewhat related to the template then both should be identified in the first stage and the combination of the two components should be even more related to the template than either individually. In this case both components would be considered to relate to the template network. This should not, however, be the case if the first component is a good fit for the network and the second merely reflects a distinct network that shows some partial overlap with the template. In this situation the Jaccard similarity coefficient would be reduced by combining the two components due to the inclusion of extra regions not present in the template.

### 2.4 A systematic approach to identifying coherent RSNs and testing their function

**Fig. 2:**
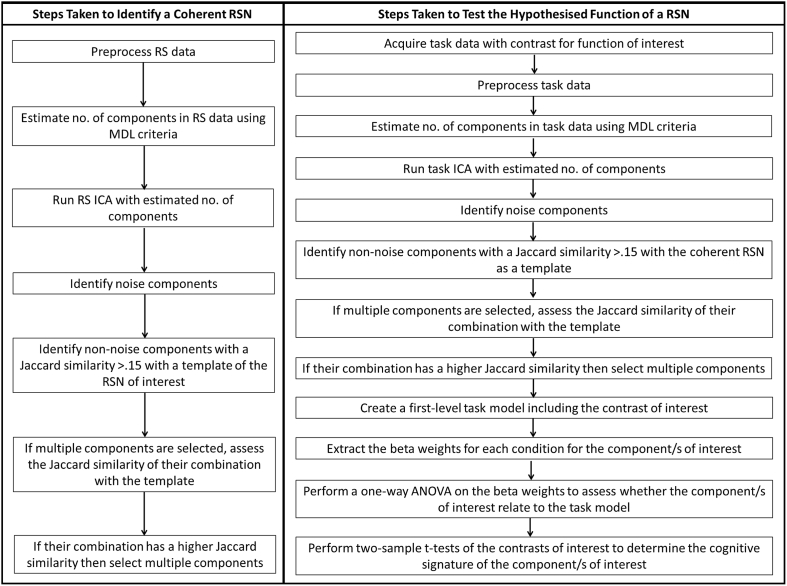
The approach used to determine coherent RSNs of interest and test their hypothesised function. MDL = Minimum Description Length. This estimation is implemented in the GIFT toolbox. For more details on the approach used, see the text.

A systematic approach was employed to separate coherent RSNs and identify those of interest (in this case the proposed semantic network and the DMN) and then to determine their function, shown in Fig. 2. Function was determined by identifying the same networks in independent task data in a separate ICA by assessing the overlap between the task components and the RSNs of interest. The advantage of identifying the network in the task data is the ability to determine the function of the spatially and temporally coherent network and not merely the constituent regions. This means that spatial overlap between networks does not affect the results unlike simple volume-of-interest analyses.

#### 2.4.1 Resting-state data

The RS scan consisted of 130 volumes collected over 6.25 min. During this time the participants were asked to lie still and fixate on a cross (Van Dijk et al., 2012). Preprocessing of the RS data was performed using statistical parametric mapping (SPM 8) software (Wellcome Trust Center for Neuroimaging) and the Data Processing Assistant for Resting State fMRI (DPARSF Advanced Edition, V2.3) toolbox (Chao-Gan & Yu-Feng, 2010). In addition, high motion time-points were identified using the ARtifact detection Tools software package (ART; www.nitrc.org/projects/artifact_detect). Preprocessing was performed as in Jackson et al. (2016) including slice time correction, realignment, coregistration, removal of motion and tissue-based regressors, normalisation using DARTEL, linear detrending and filtering. The high motion time points identified with ART were removed (‘scrubbing’) as well as removal of the 24 motion parameters [as described by Friston, Williams, Howard, Frackowiak, and Turner (1996)] and the mean activation in the CSF and white matter. These preprocessing steps greatly reduce the effect of motion and are consistent with prior research (Anderson et al., 2011, Calhoun et al., 2001, Geranmayeh et al., 2014, Power et al., 2014, Power et al., 2015, Van Dijk et al., 2012). Six participants were excluded due to high levels of motion as in the prior study (see Jackson et al., 2016). No other participants were excluded from the resting-state data. Unlike Jackson et al. (2016) removal of the global signal was not performed. Although it is still important to remove as much of the effects of motion as reasonably possible, small movements may be less of a problem for ICA than standard functional connectivity analyses as, firstly, noise components have separate sources and can be identified as separate components and secondly, the correlation between two regions is not the primary measure but whether the variance in activation in different regions may be explained by a shared source (Calhoun et al., 2001, Geranmayeh et al., 2014, Griffanti et al., 2014, McKeown and Sejnowski, 1998). Thus, the removal of the global signal is less necessary and imposing spurious negative correlations could affect the results of the ICA (Anderson et al., 2011).

#### 2.4.2 Identifying coherent resting-state networks related to the proposed semantic network and the DMN

The steps taken to identify the coherent RSNs of interest are shown in Fig. 2. In order to determine the coherent RSNs present in the RS data, ICA was performed using the Group ICA of fMRI Toolbox (GIFT; Calhoun et al., 2001). GIFT removes the mean at each time point before performing two stages of data reduction using principal component analysis (PCA; Calhoun et al., 2001). The group ICA is performed on the concatenation of each participant's PCA results. ICA assumes independent sources have been linearly combined to create the signal and attempts to separate these sources. The resulting components comprise a spatial map and a time-course of activation. Each resulting component may relate to either an artefact or neural processing or both. The resulting mixing matrix allows reconstruction of the individual participant's components. This means statistics can be performed on the individual participant's data (Calhoun et al., 2001). To obtain a group-level spatial map for each component, a one sample *t*-test was used to identify the voxels that were significantly active across individuals using the SPM-based GIFT utility. As this process results in a standard statistical map for each component, these images may be subjected to thresholding and binarisation in a typical manner. The results were thresholded at a voxel-level significance threshold of .001 and an FWE-corrected critical cluster level of .05. The number of components within the data was estimated to be 60 within GIFT using the Minimum Description Length criteria per participant and computing the mean for the group (Li, Adah, & Calhoun, 2006). Using this estimate allowed bottom-up determination of an appropriate number of components for each dataset. Only this number of components was assessed, allowing a single results space to be interpreted allowing simpler interpretation and disallowing the search of multiple results spaces for desired results.

Artefactual components were then identified and not included in any further analyses. The criterion for assigning networks as artefact was based on the spatial maps attained as a result of the one sample *t*-tests. The spatial maps were inspected both at the FWE-corrected critical cluster level of .05 (voxel-level significance of .001, as the results are presented) in order to better estimate the location of peaks and a low uncorrected threshold (t = 1.5) in order to see the full pattern of activity. Maps were visually compared to the SPM grey matter template. Components where activity was mostly outside of the grey matter (whether it be within the ventricles, white matter or outside the brain) were removed as they likely relate to motion artefact. Likewise, components focussed on the brain stem were considered likely to reflect blood flow changes and were similarly removed. Components that constituted a ring or crescent around the edge of the brain were removed as this can be caused by the realignment and normalisation preprocessing steps. Therefore, components that were retained had the majority of activity within the grey matter and were not merely the edge of the brain. At the time of removing artefactual components, the researcher was blind to the correspondence of the components with both the network templates and the task model. These features are commonly used to determine artefactual ICA components and are in good alignment with recently published guidelines (Geranmayeh et al., 2014, Griffanti et al., 2014, Griffanti et al., 2017).

Components relating to the semantic and default mode networks were then identified using the Jaccard similarity coefficient. All non-noise components were thresholded at a voxel-level significance of .001 and an FWE-corrected critical cluster level of .05 and compared to the proposed semantic network from Jackson et al. (2016) in order to determine whether there was a similar coherent network. All non-noise components were compared to the seed-based DMN shown in Fig. 1C and (independently) to a DMN template freely available online (http://findlab.stanford.edu/functional_ROIs.html; Shirer et al., 2012). This meant that the coherent DMN could be identified in a manner consistent with both the seed-based analysis (allowing comparison to the semantic network) and the literature as a whole.

### 2.5 Investigating the function of the coherent RSNs using task data

The steps taken to test the hypothesised function of the coherent RSNs of interest are shown in Fig. 2.

#### 2.5.1 Semantic task data

Task data were used to verify the separation between the RSNs of interest was meaningful and to relate them to cognition. Three runs of task data were used, each lasting 10 min and including 211 volumes. Within these runs there were three conditions; a semantic judgement task, a non-semantic baseline task and rest. Both tasks involved pressing a button to indicate which of two targets was related to a probe. For the semantic judgement this meant which word was more related in terms of meaning. For the non-semantic baseline this meant the set of letters that contained the most letters in common with a probe set. See Jackson et al. (2015) for further details including the experimental stimuli. The resting-state data were collected in the same session as task data for ease of collection. Therefore, the participants involved in the task fMRI study also underwent resting-state data collection. However, all analyses were performed on either the resting-state dataset or the task dataset and the majority of the resting-state participants had not performed the task. It is therefore not considered critical that data from the same participants were used for the two analyses. The task data underwent the same preprocessing stages as the RS data in order to reduce the effect of motion and make the results comparable. This resulted in the exclusion of one participant. No other participants or data were excluded from this study.

#### 2.5.2 Identifying the coherent networks in the task data & determining their function

ICA was performed on the task data, independently to the resting-state data. GIFT identified 92 dimensions within the task dataset. The task ICA components were classified as noise based on the spatial maps in the same way as the RS ICA components. The remaining components were binarised at a voxel-level significance threshold of .001 and an FWE-corrected critical cluster level of .05 and compared to the RSNs of interest using the Jaccard similarity coefficient in order to identify these networks (if apparent) in the task data. Like the RS analysis, spatially and temporally coherent networks could be identified. Unlike the RS ICA components, the function of the networks of interest could be assessed by comparing their time-course to the task model using the ‘Stats on Beta Weights’ function in GIFT. To do so, the group level component is back-propagated to determine the spatial map and time-course of the component for each individual participant (Calhoun et al., 2001). A design matrix was created in SPM8 for each participant including the three conditions, the semantic task, the non-semantic baseline task and rest. GIFT was used to extract the beta coefficients for each condition per component and perform a one-way ANOVA for each component, using the onsets for each condition as a factor. This showed whether the beta weights significantly depended on the condition i.e., whether the activity in that component is significantly related to the task model. For components with a significant ANOVA result, three planned contrasts were performed. The beta weights were compared using independent sample *t*-tests for the semantic task > non-semantic control task, semantic task > rest and non-semantic task > rest. Bonferroni correction was applied for the three contrasts assessed. The critical contrast of interest was the semantic task > non-semantic task. As rest may include a large amount of semantic processing, comparison between a semantic task and rest may fail to find critical regions or networks. However, a network responsible for semantics should be identifiable when contrasted with a non-semantic task. The use of a high-level baseline has been found to be critical in identification of semantic regions (Visser, Jefferies, & Lambon Ralph, 2010). Therefore, this contrast alone is used to determine whether a network is responsible for semantic cognition or not. However, the contrasts against rest are shown for each network of interest, to aid interpretation of the components function using the full pattern of results and to show how comparison to rest only may have affected the conclusions. All other non-artefactual task ICA components were assessed for significantly greater activity in the semantic task than the control condition (the main contrast of interest). Overall, all non-artefactual components are assessed for their relation to the networks of interest and their role in semantics, therefore all relevant measures, conditions and data exclusions are included in the present article.

## 3 Results

### 3.1 Identifying coherent resting-state networks-of-interest

The ICA of the RS data identified 60 components. Of these 60, 27 resting-state components were clearly attributable to artefact, leaving 33 which may relate to neural signals. The number of components identified and their spatial profiles are similar to prior resting-state studies and many appear to reflect established sensory, motor or cognitive networks (e.g., Smith et al., 2009). All are shown in Supplementary Figure 1.

**Fig. 3:**
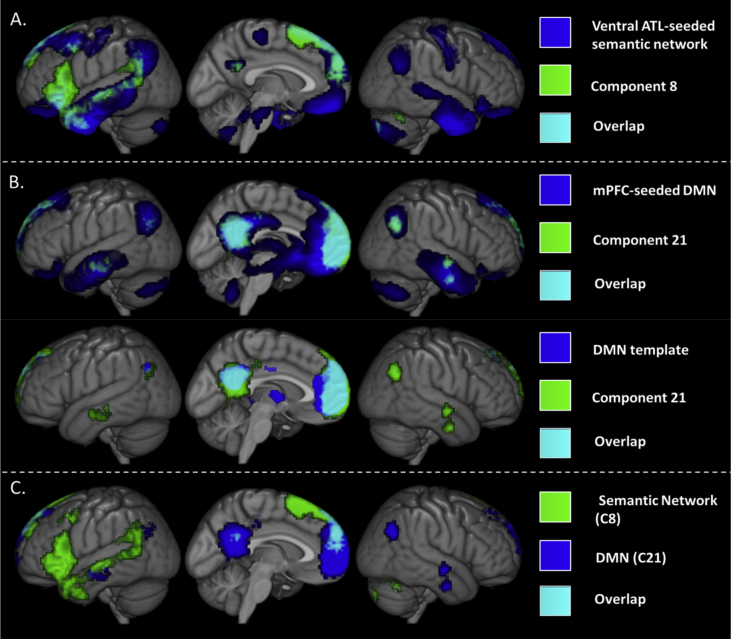
Identifying the components-of-interest in the resting-state. A. Component 8 (green) was identified as a good match spatially to the ATL-seeded network identified in the resting-state in Jackson et al. (2016) (blue). Overlap is shown in cyan. B. Component 21 (green) was identified as a good match spatially to both the mPFC-seeded default mode network (upper portion, blue) and an *a priori* DMN template (lower portion, blue). C. The two selected components are shown. The component hypothesised to be semantic is shown in green and the DMN component is shown in blue. Overlap is in cyan. Although both networks are more circumscribed than the seed-based analyses there is still some overlap, particularly within mPFC. All results have a voxel-level significance threshold of .001 and an FWE-corrected critical cluster level of .05.

All non-noise components images were binarised at a voxel-level significance threshold of .001 and an FWE-corrected critical cluster level of .05. The component corresponding to the semantic network proposed in Jackson et al. (2016) was identified in order to determine whether this is a coherent network and its spatial extent. The seed-based result (including all areas significantly functionally connected to the ventral ATL) was binarised and used as a template. All ICA components were compared to this template using the Jaccard similarity coefficient. Only one component, Component 8 (J = .166) was above the overlap threshold, shown overlaid on the ATL seed-based network in Fig. 3A. This component consisted of key semantic regions including ATL, IFG, dorsal mPFC and IPL (see Table 2) and overlapped strongly with the seed-based result. Component 8 is henceforth referred to as the proposed ‘semantic network’. Overall, the component is less extensive and shows greater bias towards the left hemisphere than the ATL seed-based FC result but involves all the same key regions.

**Table 2:** Peak activation in the resting-state components-of-interest. Voxels are significant at .001. Clusters are significant with FWE-correction and a critical cluster level of .05.

Component	Cluster extent (voxels)	Max z value	P value (FWE corrected)	Peak MNI Coordinate			Region(s)
				X	Y	Z	
8 Semantic Network	1793	>8	<.001	−48	27	−3	L IFG, TP, MTG, AG
	926	7.54	<.001	−6	12	63	L SMA,dmPFC
	105	5.84	<.001	−45	6	45	L preCG
	247	5.48	<.001	27	−78	−33	Cerebellum
	29	4.38	.012	9	−72	57	R precuneus
	29	4.13	.012	−3	−51	27	L PCC
21 Default Mode Network	2106	>8	<.001	3	57	−6	Mid mPFC
	615	>8	<.001	−3	−54	27	Precuneus, PCC
	86	5.83	<.001	−45	−66	27	L AG
	95	5.61	<.001	57	−66	30	R AG
	66	4.9	<.001	63	−6	−30	R aITG, aMTG
	43	4.66	.001	−51	0	21	L preCG
	39	4.19	.002	−66	−18	−18	L aITG, aMTG
	25	4	.029	24	−18	−12	R hippocampus
	32	3.98	.008	24	−18	60	R preCG

L = left R = right a = anterior d = dorsal IFG = inferior frontal gyrus TP = temporal pole AG = angular gyrus MTG = middle temporal gyrus SMA = supplementary motor area FG = fusiform gyrus mPFC = medial prefrontal cortex PCC = posterior cingulate cortex preCG = precentral gyrus PHG = parahippocampal gyrus ITG = inferior temporal gyrus.

All non-noise RS components were then compared to the binarised seed-based DMN result (i.e., all areas significantly functionally connected to a mPFC region commonly used to identify the DMN, shown in Fig. 1C.) in the same manner. Identifying a component corresponding to this DMN template assessed whether there was a coherent DMN in the RS data. Determining whether this was the same component as the semantic network or a distinct component determined whether the semantic network and DMN reflect one or two coherent networks. Comparison of the seeded DMN to all non-noise components identified one component above the threshold (Component 21; J = .171). In addition, comparison to an *a priori* DMN template (Shirer et al., 2012) identified only the same component (J = .405), confirming that this is the DMN. This was not the same component as the semantic network (Component 21 *vs* 8). Component 21 is shown overlaid on the seed-based DMN in Fig. 3B and the *a priori* DMN template in Fig. 3C, and is highly similar to both. This component includes mPFC, PCC, angular gyrus and medial and lateral temporal lobe (see Table 2). All regions identified within the seed-based analysis are present although the extent of the areas involved is reduced.

Both the proposed semantic network and the DMN were identified as components in the RS ICA. They were however, separate components, and therefore reflect two distinct coherent networks, one which will be referred to as the semantic network (Component 8) and one the DMN (Component 21). Both ICA components included the same general regions as the seed-based FC analyses but were more circumscribed. It should be noted that the degree of overlap between the two networks was much reduced by the use of ICA (as compared to seed-based FC analyses) yet some overlap was still present, primarily within dorsal mPFC (see Fig. 3C). The implications of this are outlined in the Discussion. Further analyses were focussed on determining the function of these two coherent networks.

### 3.2 Investigating the function of the coherent resting-state networks using a task ICA

Having identified two distinct coherent RSNs of interest it is critical to determine whether they differ in terms of their function. Not only is understanding function central to the aims of cognitive neuroscience, but this step is necessary to allow interpretation of the separation between the two networks. For example, one network could theoretically split into two ICA components on a basis that is not psychologically meaningful, for instance, when there is sufficient uncorrelated noise in different regions of a single network. However, these two components would still show a similar task relation. Accordingly, the cognitive signature of the two RSNs of interest was assessed with a focus on semantic cognition.

As noted above, the aim was to assess the function of spatially and temporally coherent networks and not merely of the constituent regions of a network. This was achieved through identification of the network in task data. A separate ICA of task data was used to identify the set of coherent networks present in this dataset. The networks-of-interest were identified in the task data based on their spatial overlap with the semantic and DMN RSNs, and then their functions were formally explored by assessing the relation of their time-course with the task model.

#### 3.2.1 Identifying the coherent networks in the task data

Of the 92 components identified within the task data, 43 were clearly attributable to artefact, leaving 49 which may relate to neural signals of interest. This is a similar proportion to the RS ICA (task ICA; 53%, RS ICA; 51%). Many of these components appear to reflect known functional networks and all are shown in Supplementary Figure 2.

**Fig. 4:**
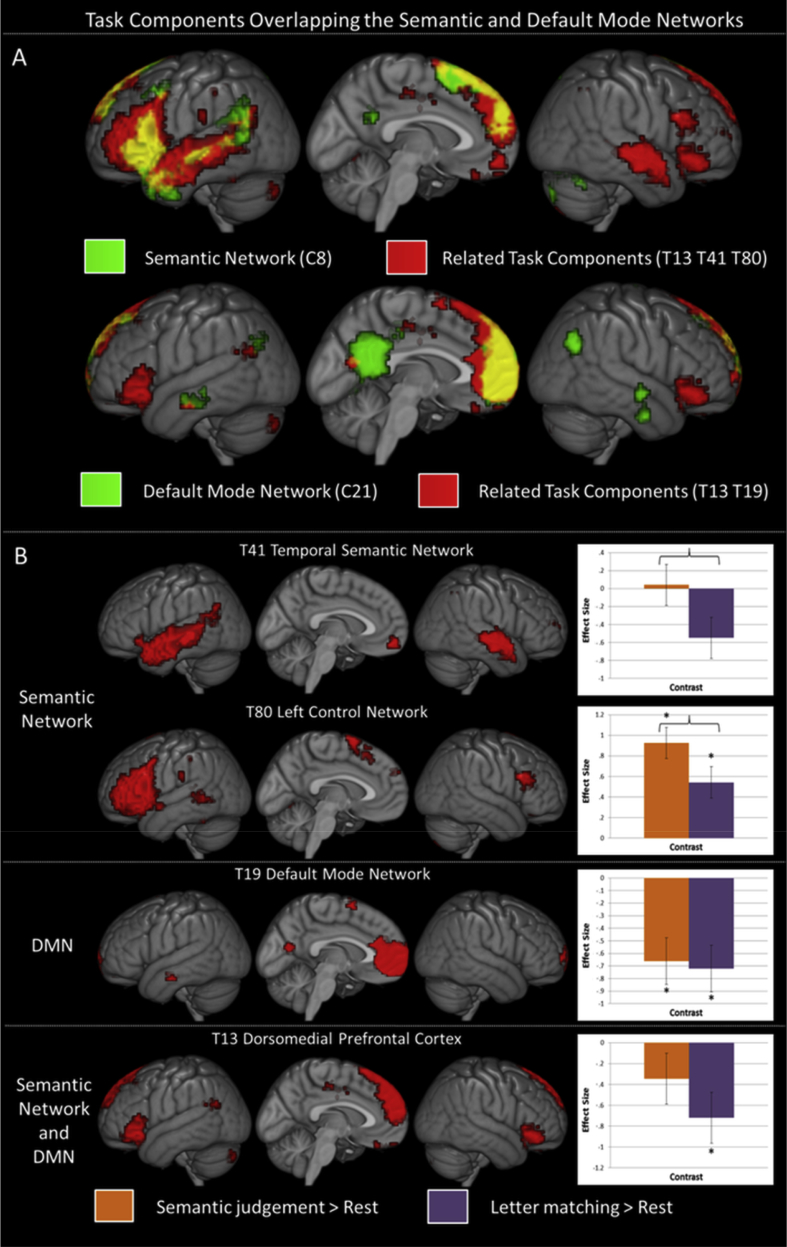
Determining the cognitive signature of the coherent RSNs using an ICA performed on independent task data. A. The components from the task data were compared to the spatial profiles of the two coherent resting-state networks-of-interest identified with ICA, the semantic network and the default mode network. This identified 3 task components relating to the semantic network (upper row) and 2 relating to the DMN (lower row). The task components are shown in red and the resting-state components used as templates in green. One of these components is the same for the two networks (T13). All are shown at a voxel-wise significance threshold of .001 FWE-corrected at the cluster level with a critical cluster level of .05. B. Each task component matching the semantic network, the default mode network or both is shown in red alongside its cognitive signature determined based on the fit to the task model. The semantic judgement task > rest is shown in orange and the letter matching baseline > rest is shown in purple. Differences significant at *p* < .05 are highlighted with an asterisk. As a substantial amount of semantic processing may occur during rest, here the critical contrast of interest is semantic judgement > control i.e., the difference between the two columns. The significance (at *p* < .05) of this difference is highlighted with a brace. Here T41 and T80 that correspond with the semantic network are found to be critical for semantics, whereas the DMN-related components are not.

The Jaccard similarity coefficient was used to compare each task component to the RSNs of interest. The semantic RSN (C8) was compared to all non-noise task components and 3 were above threshold (Task Component 13; J = .199, Task Component 80; J = .170, Task Component 41; J = .164). Combining all 3 components led to the highest overlap (Task Components 13, 80 and 41; J = .262), therefore all 3 were considered to constitute the proposed semantic network. The overlap between these task components and the semantic RSN is demonstrated in Fig. 4A. Each component is shown in Fig. 4B and activity peaks are listed in Table 3. Task Component 13 primarily consisted of bilateral dorsomedial PFC, as well as some IFG, AG and cingulate cortex (see Fig. 4B and Table 3). Task Component 41 included numerous regions in the temporal cortex (including anterior ITG and MTG as well as posterior MTG, STG and fusiform gyrus) with additional involvement of orbitofrontal cortex, dorsolateral prefrontal cortex, ventromedial prefrontal cortex and angular gyrus. Task Component 80 primarily consisted of IFG and posterior MTG, with a left hemisphere bias (see Fig. 4B and Table 3). Whilst these areas work together during rest, they may have differentiable functions which could lead to a separation into multiple components in relevant task data. Alternatively, these regions may separate due to other systematic differences, such as uncorrelated noise, but have no difference in function or veridical time-course. Overall, these three components are highly similar to the coherent semantic network identified within the RS although more right hemisphere involvement was observed (see Fig. 4A.).

**Table 3:** Peak activation in the task components-of-interest. Voxels are significant at .001. Clusters are significant using FWE-correction and a critical cluster level of .05.

Component	Relation	Cluster extent (voxels)	Max z value	P value (FWE corrected)	Peak MNI Coordinate			Region(s)
					X	Y	Z	
T41	Semantic Network	1544	7.05	<.001	−57	−9	−9	L temporal, lOFC, vAG
		702	6.53	<.001	51	−30	0	R temporal
		56	5.06	<.001	36	48	12	R DLPFC
		60	4.69	<.001	−3	54	−12	L vmPFC
		33	4.09	.002	45	−57	42	R IPL
T80	Semantic Network	1890	7.73	<.001	−45	30	12	L IFG, insula, preCG
		60	4.99	<.001	30	39	−9	R lOFC
		148	4.96	<.001	−36	−60	39	L IPL
		218	4.95	<.001	45	27	18	R IFG
		167	4.93	<.001	−60	−48	3	L pMTG, pSTG
		116	4.84	<.001	−3	6	66	SMA
		47	4.83	<.001	−48	−54	−21	L pFG, pITG
		24	4.73	.019	−9	54	30	L dmPFC
		20	4.53	.046	−63	−27	27	L SMG
		30	4.31	.005	−15	−66	−12	L occipital, cerebellum
		37	4.09	.001	9	−81	−30	Cerebellum
		21	3.67	.036	24	−69	−54	Cerebellum
T19	DMN	1479	>8	<.001	0	54	−6	Mid mPFC
		32	4.42	.004	−6	−66	15	Cuneus, precuneus
		24	4.34	.019	−63	−15	−18	L aMTG
		20	3.95	.046	−6	9	66	L SMA
T13	Both	2011	6.98	<.001	−3	51	36	dmPFC, DLPFC
		276	6.3	<.001	−48	36	−12	L IFG
		103	5.52	<.001	−30	−84	−33	Cerebellum
		110	5.35	<.001	−51	−63	24	vAG
		195	4.9	<.001	48	33	−6	R IFG, superior TP
		42	4.87	<.001	0	39	−24	mOFC
		49	4.53	<.001	3	−18	45	Mid CC
		27	4.8	.006	27	−78	−33	Cerebellum

L = left R = right a = anterior p = posterior v = ventral d = dorsal m = medial l = lateral TP = temporal pole MTG = middle temporal gyrus FG = fusiform gyrus ITG = inferior temporal gyrus AG = angular gyrus IPL = inferior parietal lobe SMA = supplementary motor area IFG = inferior frontal gyrus PFC = prefrontal cortex OFC = orbitofrontal cortex DLPFC = dorsolateral prefrontal cortex preCG = precentral gyrus CC = cingulate cortex.

The DMN was also identified in the task data through comparison of the coherent DMN (determined in the RS ICA) to all non-noise task components. This identified two related task components, one of which related to the DMN alone (T19: J = .285, see Fig. 4). This comprised core DMN regions, including mid mPFC (Brodmann areas 10 and 11), cuneus and precuneus as well as left lateral temporal cortex and supplementary motor area. The other was Task Component 13, already identified as relating to the semantic network (J = .217). The combination of these components showed greater overlap than either component individually (J = .342) so both were considered part of the DMN.

In summary, four task components were highlighted as related to the two RSNs of interest. Specifically, two task components were identified as being uniquely related to the semantic network, one uniquely to the DMN and one to both networks (T13). How this component may relate to the DMN and the proposed semantic network is considered further in the Discussion.

#### 3.2.2 Determining the function of the networks identified in the task data

The function of these four task components was investigated by extracting the beta weights associated with each condition in the task model for that component. An ANOVA was used to assess whether the components activity significantly related to the task model, consisting of semantic judgement, letter matching baseline (an active control task) and rest conditions. All components-of-interest showed a significant relation to the model (T41; F(2, 68) = 4.136, *p* = .020, T80; F(2, 68) = 18.386, *p* < .001, T19; F(2, 68) = 9.295, *p* < .001, T13; F(2, 68) = 4.353, *p* = .017). Thus, planned *t*-tests comparing the three conditions were performed (with Bonferroni correction for three comparisons) in order to identify the cognitive ‘signature’ of each component. The cognitive signature for each component-of-interest is shown in Fig. 4B.

Whether each component was responsible for general semantic processing was determined by the result of the semantic task > non-semantic task contrast. Both semantic network components showed significantly higher activation for the semantic judgement than the letter matching baseline suggesting these components are involved in semantics (semantics > letter matching; T41; t(70) = 2.578, *p* < .005, T80 t(70) = 2.495, *p* < .005, Bonferroni corrected). However, the profiles of the two components are distinct; the temporal lobe component, T41, shows a trend towards activation for the semantic condition (semantics > rest; t(70) = .184, *p* = 1, Bonferroni corrected) but a trend towards deactivation of the non-semantic task from rest (letter matching > rest; t(70) = -2.394, *p* = .060, Bonferroni corrected), whereas the component primarily comprised of IFG and pMTG, T80, shows activation of both tasks from rest (semantics > rest; t(70) = 6.034, *p* < .001, letter matching > rest; t(70) = 3.539, *p* < .05, Bonferroni corrected), yet higher involvement in the semantic condition. Thus, T41 shows the expected pattern for a network specifically involved in semantic cognition, whereas T80 shows a more task-general pattern, yet greater involvement when semantic processing is required. The relatively small difference between the semantic condition and rest for T41 may be seen as evidence that semantic representation processes are highly engaged during the resting-state. Interestingly, if the critical comparison had been to rest, T41 would not have reached significance despite including regions known to be critical for semantics in the patient literature, whilst T80 would be easily identified as semantic despite its relatively task-general profile (see Discussion).

The DMN component T19, showed no difference between the two tasks (t(70) = .324, *p* = 1, Bonferroni corrected) and therefore no evidence of a significant relation to core semantic cognition processes. This component demonstrated significant deactivation of both tasks from rest (semantics < rest; t(70) = 3.561, *p* < .05, letter matching < rest; t(70) = 3.885, *p* < .001, Bonferroni corrected). This is a different cognitive signature to the semantic network and therefore supports the assertion that the separation of the two networks is psychologically meaningful and not merely artefactual. The general pattern of deactivation found means that this component would not be considered semantic whether the critical comparison was to an active baseline or to rest. The task component related to both semantic and DMNs (T13) also showed no significant difference between the semantic and non-semantic tasks (semantics > letter matching; t(70) = 1.538, *p* = .387, Bonferroni corrected) and is therefore not considered to be involved in the semantic task. This component showed significant deactivation for the non-semantic task and non-significant deactivation for the semantic task (semantics < rest; t(70) = 2.95, *p* < .05, letter matching < rest; t(70) = 1.412, *p* = .488, Bonferroni corrected). No relation between the semantic cognition task and any task component related to the DMN could be identified. Demonstrating differing cognitive profiles between the semantic network and DMN components confirms that these networks have been separated in a functionally meaningful manner (and not for instance, merely due to artefact) and can therefore be considered as distinct networks.

Although the main aim of the paper was to identify the function of the resting-state networks of interest, assessing the relation of all task components to the task model could allow identification of other networks responsible for semantics. Therefore, all non-noise components found to have a significant relation to the model were compared on the main condition of interest, semantics > control. This contrast was not significant for any component (*p* > .05) therefore no additional networks are argued to be responsible for semantic cognition.

## 4 Discussion

For resting-state (RS) connectivity research to inform cognitive neuroscience, we need formal methods to determine the relationship between coherent RSNs and their cognitive functions. To help demonstrate how researchers can do this, we set out a series of logical steps to identify coherent RSNs and formally determine their cognitive signature. When applied to our test-case cognitive domain, this allowed demonstration of a coherent RSN responsible for general semantic cognition, distinct from the DMN (which, itself, exhibited no relationship to the kind of semantic processing performed in the task). Whilst the precise function of the DMN is not delineated in the current study, establishing that the two networks have a differentiable relationship to general semantic cognition is sufficient to demonstrate that their split is meaningful. We will consider the specific test-case exemplar presented here and its implications for semantic cognition and then the potential for the current approach more generally.

The proposed semantic network and DMN were identified as two distinct coherent RSNs. These ICA-based networks involved the same core regions as the seed-based FC results (allowing identification using the *a priori* templates), yet involved more circumscribed areas. The semantic network encompassed ATL, pMTG, IPL, IFG, mPFC. The DMN comprised core mPFC and PCC regions, as well as angular gyrus, and medial and lateral temporal lobe, fitting the prior literature extremely well (Buckner et al., 2008, Greicius et al., 2003, Jackson et al., 2016). Thus, unlike the extensive, highly overlapping seed-based results, the two coherent networks had very different spatial extents, including distinct but nearby temporal and IPL regions reflecting known functional subdivisions (Buckner et al., 2008, Humphreys et al., 2015, Humphreys and Lambon Ralph, 2014, Lambon Ralph, 2014, Seghier et al., 2010). Overlap remained in the mPFC but the subregions involved were more distinct (dorsal *vs* mid mPFC). Therefore, the DMN and semantic network have distinct profiles both temporally and spatially, but were inadequately separated with simple seed-based FC analyses, resulting in an inaccurate representation of their spatial topography. This may be due to any of the proposed issues with seed-based FC analyses laid out in the Introduction.

The separation of the DMN and semantic network is meaningful as it reflects dissociable cognitive signatures. The function of the coherent spatiotemporal networks was assessed by identification of the networks in the active task data. Two task components were identified that reflected only the proposed semantic RSN, one that reflected the DMN only and one that related to both RSNs of interest. Only the two components that related to the semantic network alone (a temporal network, T41, and IFG, pMTG and supplementary motor area, T80) were involved in a task assessing general semantic cognition. Although very similar, the semantic network identified in the task data was more extensive in both left and right hemispheres, likely reflecting the greater proportion of difficult semantic processing in this dataset. The identification of two different semantic network components could reflect a functional difference related to the specific task demands. Indeed, the two task components appeared to show somewhat different cognitive signatures, despite both being involved in semantics. The temporal lobe component was involved in the semantic task only, whereas the frontal component seemed to be involved in both tasks albeit to a different extent. These areas are consistent with a distinction between semantic representation (dependent on the temporal lobe) and the controlled selection and manipulation of semantic information (reliant on IFG and pMTG; Davey et al., 2016, Jefferies, 2013). These control regions primarily work in concert with semantic areas but may also support demanding processes in other domains, such as phonology and orthography, which may explain their more task-general role (Gough et al., 2005, Vigneau et al., 2006). The current results are consistent with a semantic control network previously hypothesised on the basis of seed-based analyses (Davey et al., 2016, Vatansever et al., 2017). The consistency and functional significance of the split within the semantic network should be explored further using similar methods. The temporal lobe component would have been missed if an active baseline was not used for comparison, suggesting that semantic representation areas are highly engaged during rest, whereas control regions may be more critical in task contexts. This supports the hypothesis that a large amount of semantic processing occurs within the resting-state, yet suggests this depends upon the action of the semantic network and not the DMN. The hypothesised role of the proposed semantic network in general semantic cognition was supported.

No evidence was found for the involvement of the DMN in semantic cognition. The DMN deactivated equivalently during the semantic task and non-semantic tasks. This conflicts with the results of previous univariate analyses showing reduced deactivation for semantic tasks or overlapping regions deactivating for non-semantic tasks and activating for semantic tasks (Binder et al., 1999, Binder et al., 2009, Seghier and Price, 2012, Shapira-Lichter et al., 2013). However, univariate analyses are not capable of accounting for the known overlap between the DMN and other networks, including the semantic network, nor are they capable of delineating the edge between the nearby regions constituting these distinct networks. By assessing the cognitive signature of the coherent DMN for the first time we avoided the effect of overlapping networks and showed no relation between the DMN and task semantics. It is critical to remember that the current test case is only sufficient to investigate the role of the DMN in general semantics and more precise hypotheses may be advanced relating the network to specific semantic subprocesses. For instance, it may be that there are additional processes critical during rest that require complex combinations of concepts, such as combinatorial or sentential semantics (Binder et al., 2009, Humphries et al., 2007). Other processes may necessitate the involvement of semantics with other domains or specific types of concepts, such as social items (Irish and Piguet, 2013, Mars et al., 2012, Price et al., 2015). These processes may or may not require the DMN. Using the current method, it is possible for researchers to assess these relatively specific hypotheses to identify the exact role of the DMN in future studies. Only by precisely specifying and then testing hypotheses can the function of the DMN and other RSNs become known.

One task component (T13) overlapped with both the semantic network and the DMN (see Fig. 4A). This dorsomedial PFC component was not involved in semantic cognition. It may be that some of the variance in the dorsomedial PFC time-course may be best explained as a component separate from both the semantic and DMNs. The dorsomedial PFC has been linked to ‘unconstrained’ semantic processing (Binder et al., 2009) and domain general control processes (Fedorenko et al., 2013, Noonan et al., 2013). Indeed, the topography of this component suggests it may relate to the salience network (Seeley et al., 2007). Alternatively, the separation of this component may reflect noise and not a meaningful functional distinction. Further investigation of the connectivity of the dorsomedial PFC may allow interpretation of its function.

The semantic and DMNs are distinct coherent RSNs with dissociable functions. This is highly consistent with the observation that semantic regions deactivate for non-semantic tasks, yet core DMN regions, deactivate in both semantic and non-semantic tasks (Humphreys et al., 2015). The current model-free technique extends this work by identifying the precise spatial extent of the two networks, taking into consideration spatial overlap. The deactivation of both semantic and DMN regions during non-semantic processing may lead to a correlation between the networks despite their distinct functional roles (Humphreys et al., 2015). This correlation will be increased by motion as the networks involve many nearby regions (Power et al., 2014, Power et al., 2015, Van Dijk et al., 2012) and will result in over-extensive seed-based FC results. An alternate description of the results would be that multiple task-negative subnetworks constitute the DMN, including the semantic network and the ‘core’ DMN (Andrews-Hanna et al., 2010). This is a valid conceptualisation of the current results. However, it is unclear how much this hierarchical understanding contributes if the subnetworks perform distinct functions. Critically, the use of a single well-known label for a host of networks responsible for distinguishable processes may not aid communication of important results. An understanding of the functional role of other subnetworks, in particular the core default mode regions, may help determine the best way to conceptualise the relationship between these networks.

The current methodology allowed the identification of coherent RSNs and determination of their cognitive signature through the coordinated use of both RS and active task data. The first step was to split the resting-state data into distinct components using a high-dimensional ICA. The Jaccard similarity metric was used to systematically identify the RSNs of interest in the resulting ICA components based on comparison with *a priori* templates and seed-based results. An ICA was performed on independent data including a semantic task and an active baseline, and the task components related to the RSNs of interest identified by comparison to the RS ICA components. The relation of these components with the task model could then be assessed to determine the functional significance of their separation and test their hypothesised relations to cognitive domains.

The use of ICA was shown to be critical as distinct coherent networks could be identified in the RS data, unlike in the seed-based FC analyses. This highlights the inadequacies of seed-based analysis and suggests researchers should identify coherent networks with ICA, as suggested previously (Beckmann et al., 2005, Calhoun et al., 2001, Griffanti et al., 2014). Estimating the dimensionality for each ICA allows the use of an appropriate number of components for that dataset without the need to search multiple results spaces, which would disallow a simple interpretation. Use of the Jaccard similarity metric quantitatively demonstrated the identity of the resting-state and task components in order to reduce researcher bias. Adopting a similar approach may help researchers avoid the overwhelming prospect of interpreting each of the many components identified in an ICA, promoting functional interpretation and therefore reducing the detachment from function often found in RS studies. Determining the cognitive signature of a network is critical to show that separations are meaningful, as well as to inform cognitive neuroscience. The function of the spatiotemporally coherent network (and not simply constituent areas which may be involved in multiple networks) can be formally determined if the network of interest is identified in task data using ICA. This may be advantageous compared to assessing the activity of constituent regions (e.g., Laird et al., 2009) due to the involvement of regions in multiple networks. Whilst the combination of networks involved in an activity map can be estimated using spatial linear regression (Connolly et al., 2016), this may lack precision compared to splitting the task data into distinct components and does not allow full assessment of the cognitive signature of each network involved. Comparison of the spatial extent of a network to activity maps is inadequate to determine its function. The present results demonstrate that the overlap between the DMN and areas identified in a meta-analysis of semantic cognition (e.g., Binder et al., 2009) is insufficient to confirm a functional role of the DMN in semantics. Formal testing of the hypothesised role of a RSN is critical. Contrasting a networks activity between a task and the resting-state is not sufficient to understand its role. Any task may appear similar to rest due to periods of inactivity, off-task thoughts during the task of interest, or domain-general processing. However, processes that occur frequently during rest will exacerbate this similarity and may result in the inability to identify networks critical for this function. An active baseline provides a known entity with which to compare the task-of-interest, allowing greater interpretation (Frackowiak, 1991).

In order to use this approach, researchers would need data obtained during a well-executed task with an appropriate baseline, as well as an appropriate template for their RSN of interest. Many researchers will have task data in their domain of interest that can be reused or access to freely available task datasets, such as those provided as part of the Human Connectome Project (Van Essen et al., 2013). The dataset should possess sufficient power to allow separation in to a high number of dimensions otherwise RSNs responsible for distinct cognitive domains yet with correlated timecourses may not be well separated. Templates for well-known RSNs are freely available, whilst novel RSNs can be assessed using seed-based methods in order to generate templates prior to the ICA. The flexibility of the template overlapping steps outlined here mean that templates do not have to perfectly match the component to be identified, therefore the ‘best guess’ at a RSN may be used. Overall, these requirements are not likely to be too restrictive. It is possible that a RSN of interest may not be identifiable in some task data due to state-dependent connectivity changes, although this may suggest that the RSN is not responsible for a key process in this task. Additionally, a RSN could be responsible for a process that is not typically studied with task fMRI. In this case this approach may be used to reject other hypotheses, perhaps allowing future hypothesis generation and the creation of more applicable task fMRI datasets.

The steps used here to identify coherent RSNs and determine their cognitive signature are described in detail, in order that other researchers are able to use the same approach to determine the function of RSNs of interest to them. Unlike much prior research (e.g., Bertolero et al., 2015, Bzdok et al., 2016, Cole et al., 2014, Crossley et al., 2013, Krienen et al., 2014, Laird et al., 2011, Smith et al., 2009, Yeo et al., 2015) showing a general similarity between networks identified in rest and task states, the steps presented here are designed to allow a simple assessment of the function of a specific RSN of interest. Identifying a set of RSNs and their corresponding functions would be extremely fruitful for future research, for instance, allowing greater interpretation of the changes in resting-state connectivity induced in disease states. In particular, the same method could be used to test further predictions regarding the role of the DMN; assessing whether it is responsible for a specific form of semantic processing, such as social semantics, or processing within a different domain, such as episodic cognition, or whether it may be more reasonable to assess its role in forms of cognitive control that could be crucial during rest (Bar, 2009, Crittenden et al., 2015).

## Declarations of interest

None.
