# Supplementary material for: Exploring distinct default mode and semantic networks using a systematic ICA approach
Source: Cortex. 2019 Apr;113:279–97. doi: 10.1016/j.cortex.2018.12.019 (PMC6459395; doi:10.1016/j.cortex.2018.12.019)
Supplement: Multimedia component 1 [file mmc1.docx]

**A systematic approach to formally test the function of a coherent resting-state network using ICA**

Rebecca L. Jackson, Lauren L. Cloutman & Matthew A. Lambon Ralph

**Supplementary Information**


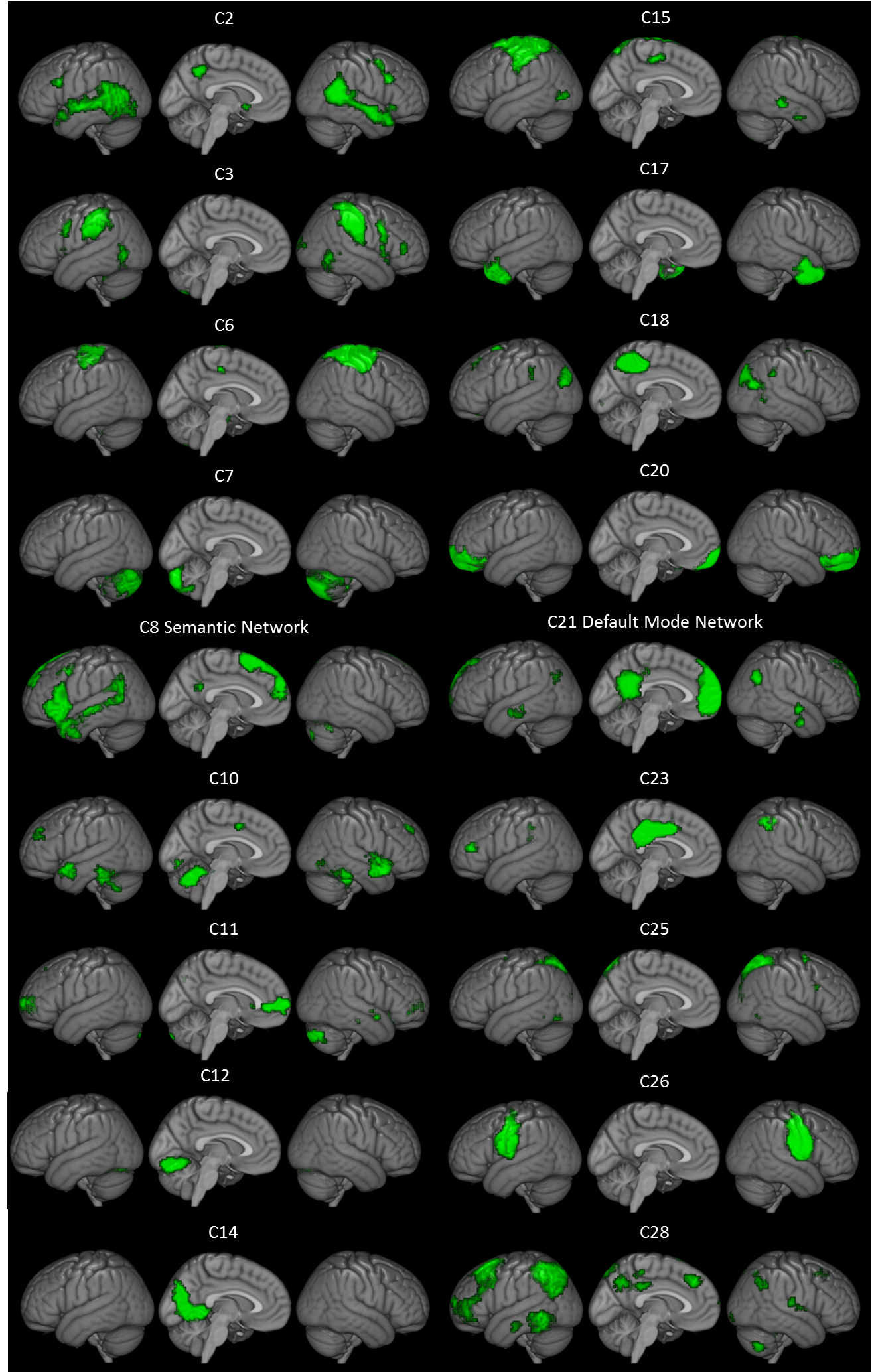


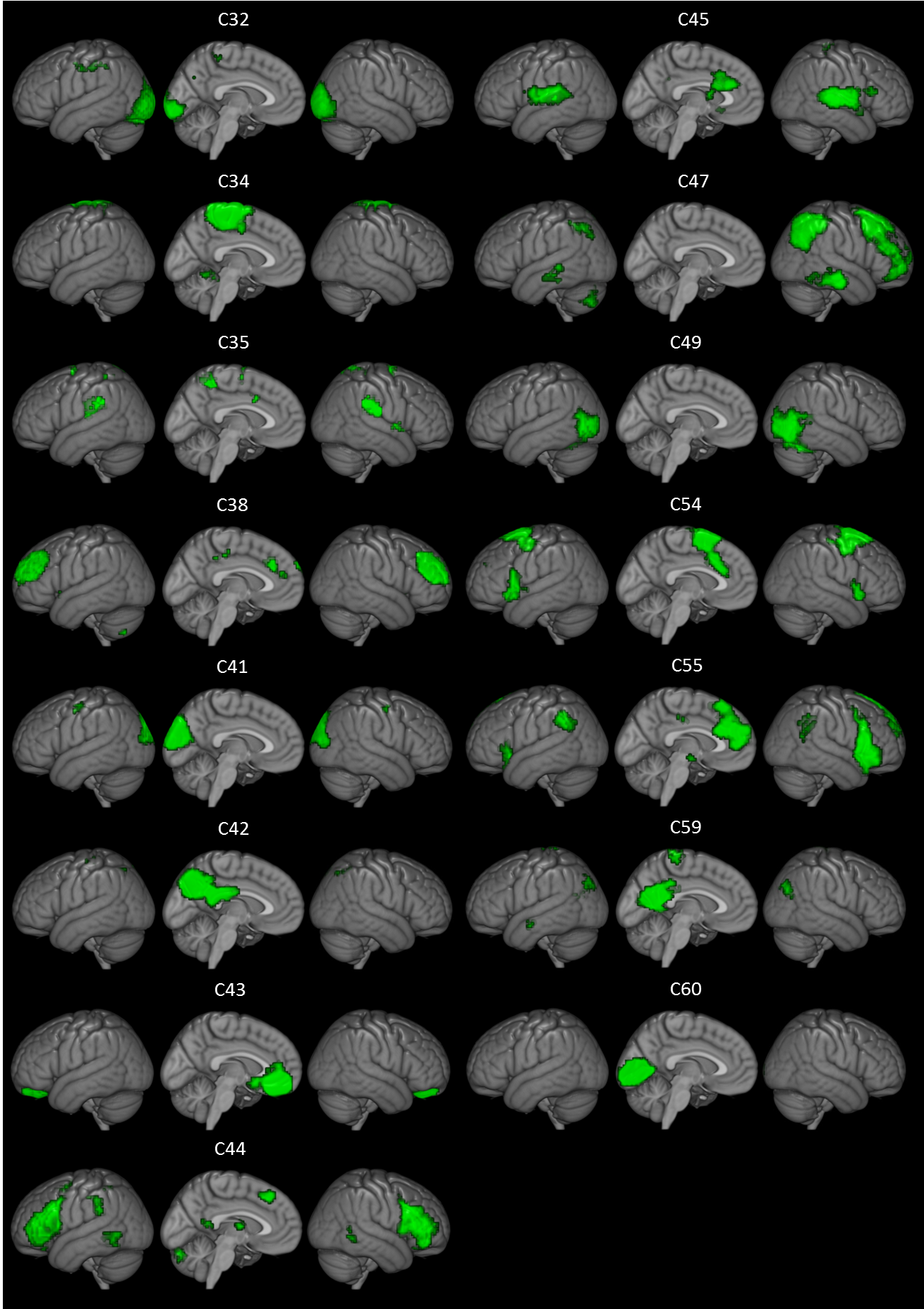


*Supplementary Figure 1.* *All non-noise resting-state ICA components.* *Components are labelled by number only unless they have been formally identified as related to a network of interest.*


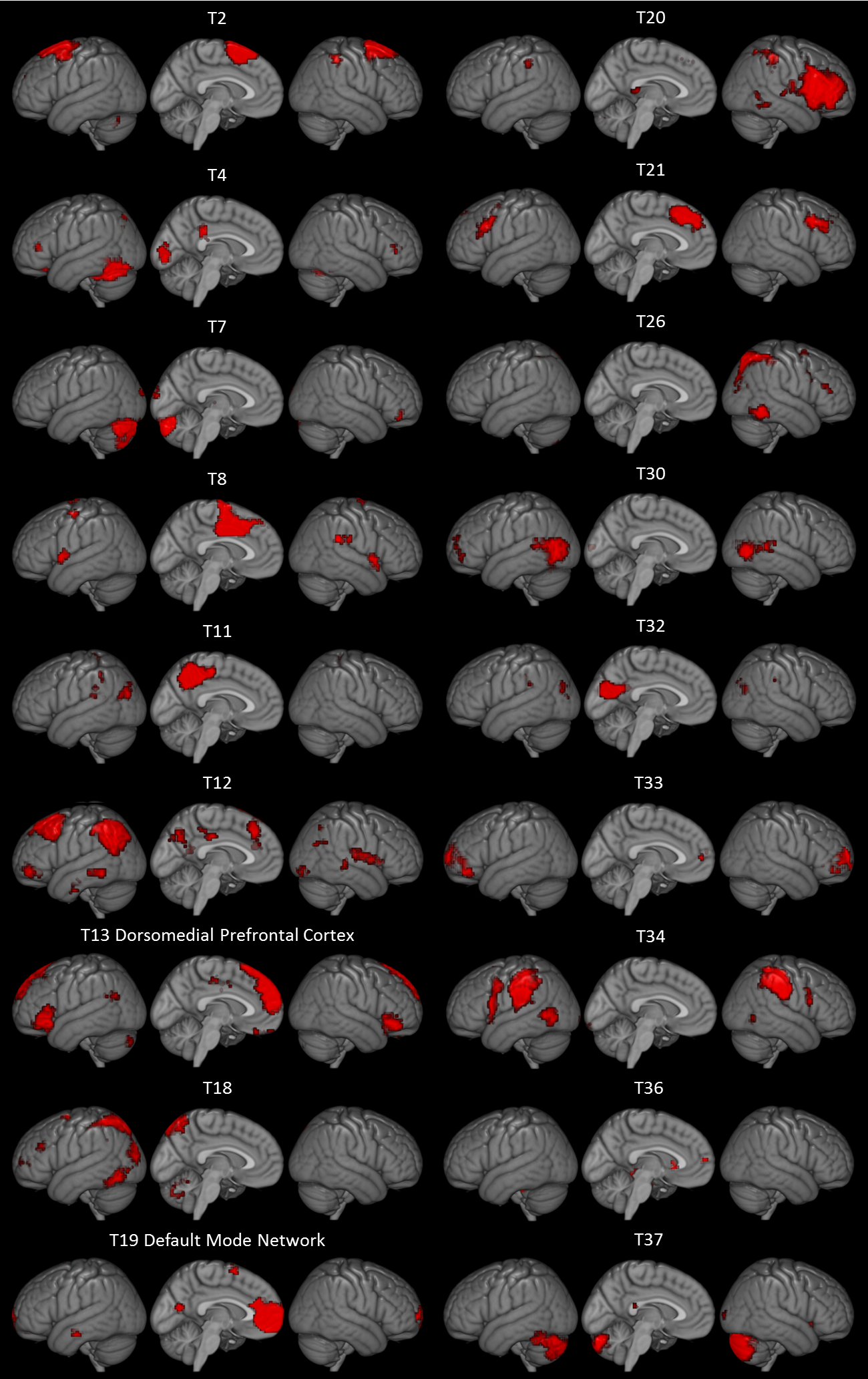


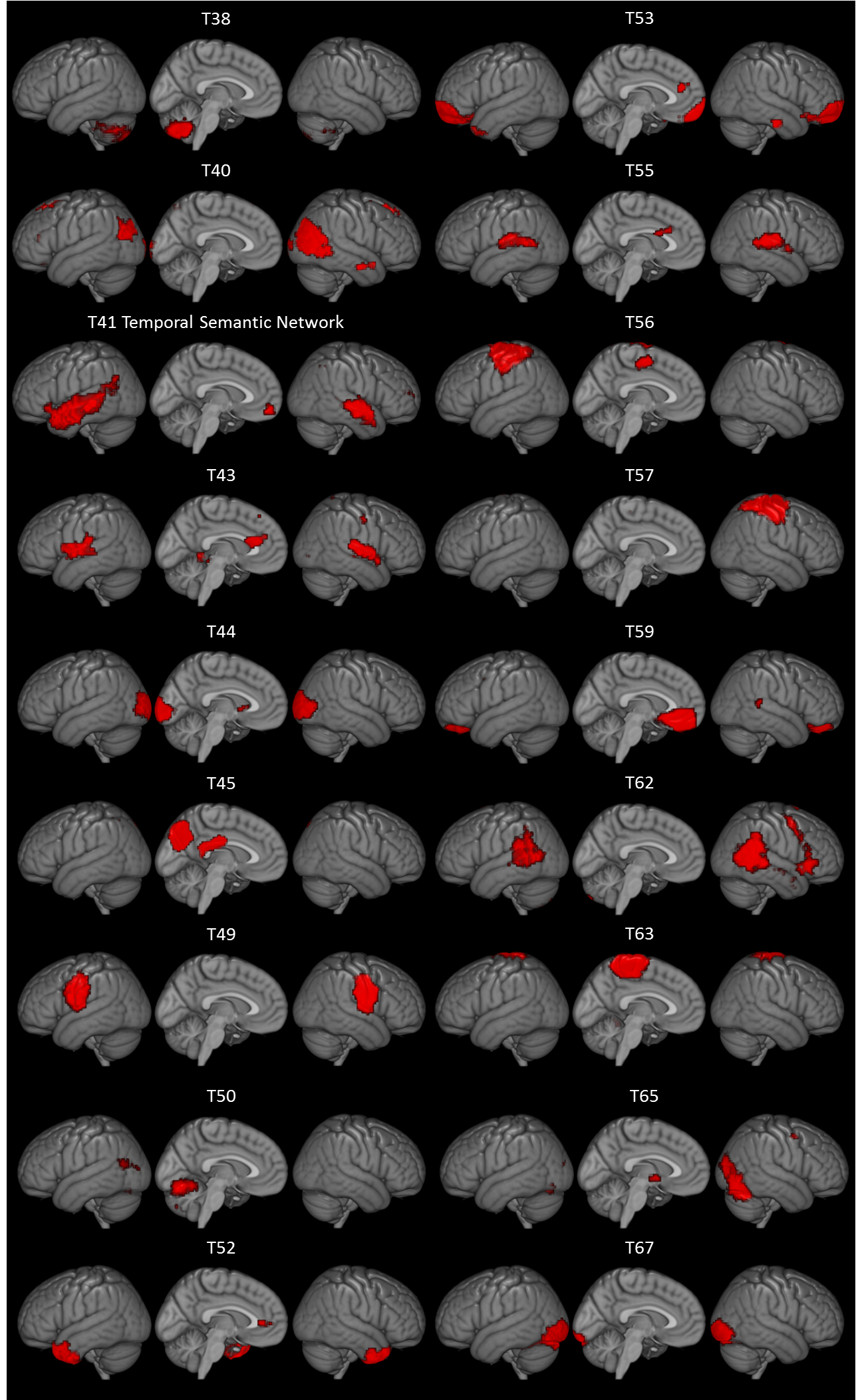


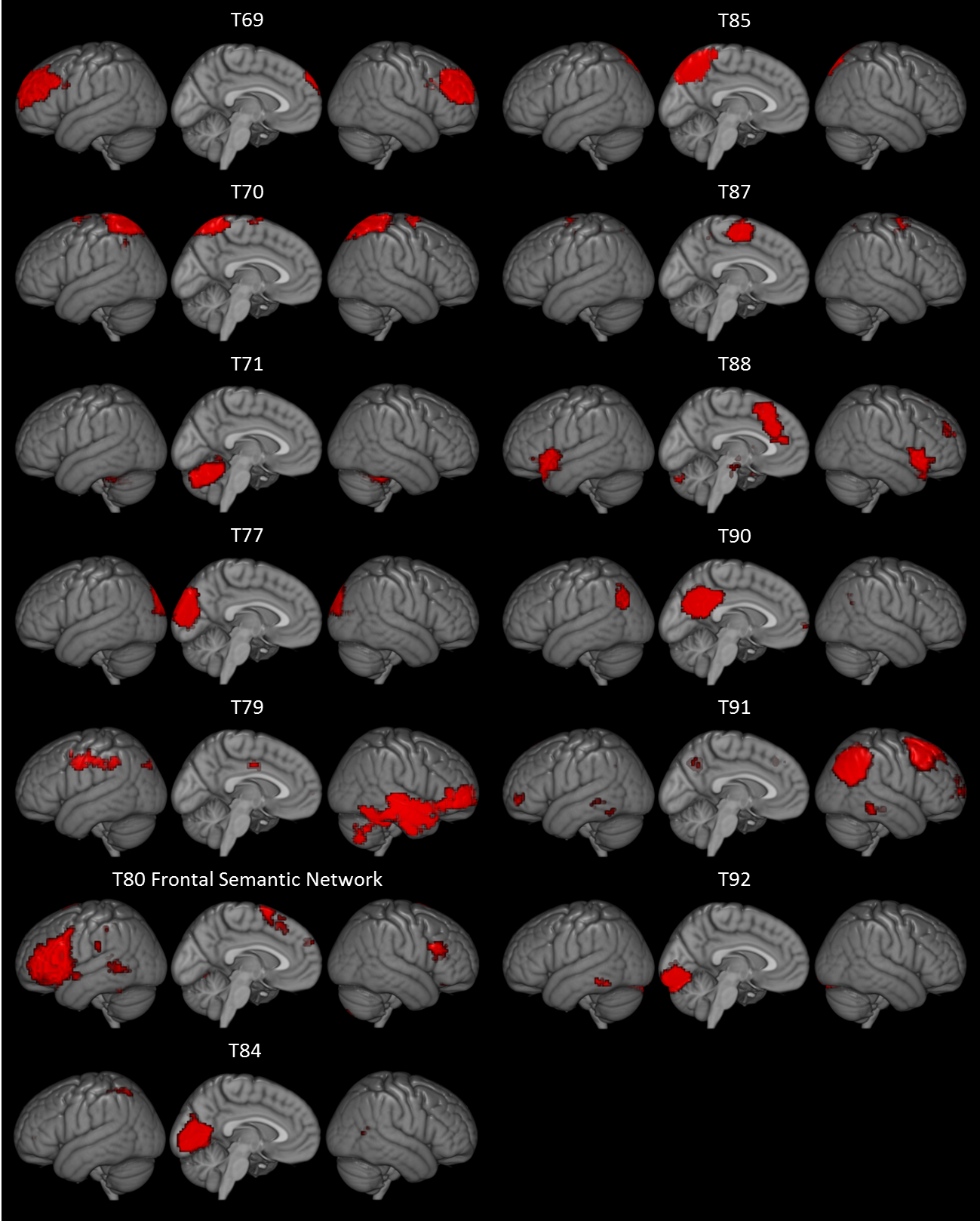


*Supplementary Figure 2.* *All non-noise task ICA components. Components are labelled by number only unless they have been formally identified as related to a network of interest.*

Supplementary Code 1.

%%Matlab script to compute Jaccard similarity coefficient between one or %%more template images and ICA component results. Written by Claude Bajada %%and Becky Jackson.

clc;clear;

template_folder = uigetdir('','Please select folder containing all template images');

results_folder = uigetdir('','Please select folder containing all results images');

output_folder = uigetdir('','Please select folder to save output matrix');

templates = cellstr(ls(strcat(template_folder, '\*.img')));

results_data = cellstr(ls(strcat(results_folder, '\*.img')));

my_similarity_matrix_comp = zeros( length(templates) , length(results_data) );

for i = 1 : length(templates)

%% Jaccard similarity (i.e. similarity of voxels that are involved in either component)

template_image = extract_read_image(strcat(template_folder, '\', templates{i}));

bin_template_image = +logical(template_image);

for j = 1 : length(results_data)

results_image = extract_read_image(strcat(results_folder,'\', results_data{j}));

bin_results_image = +logical(results_image);

comp_dif = abs(bin_template_image - bin_results_image);

comp_dif_sum = sum(sum(sum(comp_dif)));

comp_dif_total = sum(sum(sum(+logical(bin_template_image + bin_results_image))));

comp_dif_pcnt = 1 - (comp_dif_sum / comp_dif_total);

my_similarity_matrix_comp(i,j) = comp_dif_pcnt;

end

end

%add labels to results matrix

my_similarity_matrix_comp_labelled = horzcat(templates, num2cell(my_similarity_matrix_comp));

e = zeros(length(results_data)+1, 1);

e=num2cell(e);

for i =2:(length(results_data)+1);

e(i, 1) = results_data(i-1, 1);

end

e=e';

my_similarity_matrix_comp_labelled=vertcat(e,my_similarity_matrix_comp_labelled);

%save results matrix

cd(output_folder)

save my_similarity_matrix_comp_labelled.mat, my_similarity_matrix_comp_labelled

clearvars -except my_similarity_matrix_comp_labelled

%% extract_read_image function

function [ image, header ] = extract_read_image( image_name )

%EXTRACT_READ_IMAGE A function that uses the spm functions to read in a

%header and an image but wraps both functions up into one

% [ image, header ] = extract_read_image( image_name )

header = spm_vol(image_name);

image = spm_read_vols(header);

end
